# Supplementary material for: Risk factors and etiology of neonatal sepsis after hospital delivery: A case-control study in a tertiary care hospital of Rajshahi, Bangladesh
Source: PLoS One. 2020 Nov 13;15(11):e0242275. doi: 10.1371/journal.pone.0242275 (PMC7665583; doi:10.1371/journal.pone.0242275)
Supplement: Attachment — Submitted filename: Comments to manuscript PONE-D-20-13278.pdf [file pone.0242275.s002.pdf]

The study evaluates risk factors and etiology of neonatal sepsis, is well designed, but there is a major point needing to be clarified

MAJOR COMMENTS

Diagnosis of neonatal sepsis was based on positive blood culture, in this sense is important to describe the sensitivity and specificity of the of the diagnosis assay.

Although Blood culture assays is considered a gold standard, it has showed limited sensitivity for neonatal sepsis diagnosis, especially in low birth weight premature neonates in who its difficult to draw 1 ml blood due to their weight and total blood body volume; how does this explain?

Table 5 show a descriptive analysis, it would be desirable to include bivariate and multiregression analysis if applicable for causal bacteria and EONS or LONS sepsis

In the multidrug-resistant analysis authors report high prevalence of MDR multidrug-organisms; however, the MDR was no characterized on molecular based assay as the authors describe in the limitations statement. Therefore, its recommends to take the results with caution. Please explain the concern and resolve.

MINOR COMMENTS

Mistakes in the bibliographic references

There are some mistakes in the bibliography; in the introduction in page 5 line 94 reference appear as “?”. Please check and resolve.

Is desirable to refer the international consensus for definition of sepsis: Goldstein B, Giroir B, Randolph A. International pediatric sepsis consensus conference: Definitions for sepsis and organ dysfunction in pediatrics. Pediatr Crit Care Med 2005; 6: 2-8.
