# Supplementary material for: Risk factors and etiology of neonatal sepsis after hospital delivery: A case-control study in a tertiary care hospital of Rajshahi, Bangladesh
Source: PLoS One. 2020 Nov 13;15(11):e0242275. doi: 10.1371/journal.pone.0242275 (PMC7665583; doi:10.1371/journal.pone.0242275)
Supplement: Attachment — Submitted filename: Response to Reviewers.docx [file pone.0242275.s003.docx]

**Journal Name:** PLOS ONE

**Tracking No. (Manuscript ID):** PONE-D-20-13278

**Manuscript Title:** " Risk factors and etiology of neonatal sepsis after hospital delivery: a case-control study in a tertiary care hospital of Rajshahi, Bangladesh"

Dear Editor

Thank you very much for providing reviewer’s comments on our manuscript. We have modified and revised the manuscript accordingly, and detailed point–by-point corrections are given below:

**Response to reviewer comments:**
Reviewer #1: The study evaluates risk factors and etiology of neonatal sepsis, is well designed, but there is a major point needing to be clarified.

**Response to Reviewer Comments:** Thank you very much for your comments on our manuscript.

MAJOR COMMENTS

Diagnosis of neonatal sepsis was based on positive blood culture, in this sense is important to describe the sensitivity and specificity of the of the diagnosis assay.

**Response to Reviewer Comments:** We have described this issue with reference in Page 8 Line 158-159. [Manuscript with Track Changes].

Although Blood culture assays is considered a gold standard, it has showed limited sensitivity for neonatal sepsis diagnosis, especially in low birth weight premature neonates in who its difficult to draw 1 ml blood due to their weight and total blood body volume; how does this explain?

**Response to Reviewer Comments:** We have described and explained the issue in Page 8, Line 152-157. [Manuscript with Track Changes].

Table 5 show a descriptive analysis, it would be desirable to include bivariate and multi regression analysis if applicable for causal bacteria and EONS or LONS sepsis

**Response to Reviewer Comments:** Thank you very much for your comments, Table 5 shows the frequency of each category of mode of onset, gestational age and birth weight for different organisms such as Escherichia coli, Klebsiella pneumoniae, etc. So, we think there are no scope to apply bivariate and multiple regression analysis in here.

In the multidrug-resistant analysis authors report high prevalence of MDR multidrug-organisms; however, the MDR was no characterized on molecular based assay as the authors describe in the limitations statement. Therefore, its recommends to take the results with caution. Please explain the concern and resolve.

**Response to Reviewer Comments:** The concern was briefly discussed in Page 21, Line 342-344. [Manuscript with Track Changes]

MINOR COMMENTS

Mistakes in the bibliographic references

There are some mistakes in the bibliography; in the introduction in page 5 line 94 reference appear as “?”. Please check and resolve.

**Response to Reviewer Comments:** We have checked and made correction [Page 5 Line 94]. [Manuscript with Track Changes]

Is desirable to refer the international consensus for definition of sepsis: Goldstein B, Giroir B, Randolph A. International pediatric sepsis consensus conference: Definitions for sepsis and organ dysfunction in pediatrics. Pediatr Crit Care Med 2005; 6: 2-8.

**Response to Reviewer Comments:** According to your suggestions, we have revised this issue in Page 7-8 Line 138-146. [Manuscript with Track Changes]

Reviewer #2:

1) Prof-reading preferably by a native speaker

**Response to Reviewer Comments:** Thank you for your comments on our manuscript. We have tried with our best to make correction in English throughout the manuscript.

2) Introduction: INTRODUCTION : line 95-96:the cited study is not clear, there is no relation with the sentence just before and after it.

**Response to Reviewer Comments:** We have made correction [Page 5 Line 94]. [Manuscript with Track Changes]

METHODS: Line 114 Expectant mothers are diagnosed to have UTI based on wcc count in the urine .As cultures are the gold standard to diagnose UTI ,would you regard everyone with sterile pyuria to have UTI?

**Response to Reviewer Comments:** We described this issue in Page 6 Line 115-118 and in Page 21, Line 337-340. [Manuscript with Track Changes]

121: IMCI should be replace with IMNCI

**Response to Reviewer Comments:** We have made correction [Page 7 Line 125]. [Manuscript with Track Changes]

124:What was the indication of admission for control neonates

**Response to Reviewer Comments:** The indication of admission for control neonates has been mentioned in Page 7 Line 128-129. [Manuscript with Track Changes]

Discussion: Please mention the protocol regarding the use of first, second and third line antibiotics used to treat neonatal sepsis in your centre, if any. If no any such protocol what is the current most common empricial antibiotic used in a case of suspected sepsis at the time of admission?

**Response to Reviewer Comments:** We have discussed about this issue in Page 20 Line 324-328. [Manuscript with Track Changes]

We would like to thank the reviewers for the valuable comments. We have revised the documents to the best of our ability, but we will definitely be happy to provide further improvement if there are further clarifications required.

With best regards

Dr. Md. Golam Hossain

Professor of Health Research Group

Department of Statistics, University of Rajshahi

Rajshahi-6205, Bangladesh

E-mail: hossain95@yahoo.com
