# Supplementary material for: Risk factors and etiology of neonatal sepsis after hospital delivery: A case-control study in a tertiary care hospital of Rajshahi, Bangladesh
Source: PLoS One. 2020 Nov 13;15(11):e0242275. doi: 10.1371/journal.pone.0242275 (PMC7665583; doi:10.1371/journal.pone.0242275)
Supplement: Attachment — Submitted filename: Response to Reviewers.docx [file pone.0242275.s004.docx]

**Journal Name:** PLOS ONE

**Manuscript ID:** PONE-D-20-13278R1

**Manuscript Title:** “Risk factors and etiology of neonatal sepsis after hospital delivery: a case-control study in a tertiary care hospital of Rajshahi, Bangladesh”

Dear Editor

Thank you very much for providing reviewer’s comments on our manuscript. We have modified and revised the manuscript accordingly, and detailed point–by-point corrections are given below:

**Response to reviewer comments:**

**Reviewer #2:**

There are still some errors in English language at some places so please try to be as accurate as possible.

**Response to Reviewer Comments:** Thank you very much for your comments on our manuscript. We have revised and tried our best to improve English language of the manuscript.

What combination of two antibiotics would you recommend to start as an empirical therapy in a case of suspected sepsis as concluded from this study as opposed to the existing ceftriaxone and amikacin?

**Response to Reviewer Comments:** We have discussed about this issue in Page, 20; Line: 328-329.
We would like to thank the reviewers for the valuable comments. We have revised the documents to the best of our ability, but we will definitely be happy to provide further improvement if there are further clarifications required.

With best regards

Dr. Md. Golam Hossain

Professor of Health Research Group

Department of Statistics, University of Rajshahi

Rajshahi-6205, Bangladesh

E-mail: hossain95@yahoo.com
